# Actin Nemaline Myopathy Mouse Reproduces Disease, Suggests Other Actin Disease Phenotypes and Provides Cautionary Note on Muscle Transgene Expression

**DOI:** 10.1371/journal.pone.0028699

**Published:** 2011-12-09

**Authors:** Gianina Ravenscroft, Connie Jackaman, Caroline A. Sewry, Elyshia McNamara, Sarah E. Squire, Allyson C. Potter, John Papadimitriou, Lisa M. Griffiths, Anthony J. Bakker, Kay E. Davies, Nigel G. Laing, Kristen J. Nowak

**Affiliations:** 1 Centre for Medical Research, The University of Western Australia, Western Australian Institute for Medical Research, Nedlands, Australia; 2 Physiology, School of Biomedical, Biomolecular and Chemical Sciences, The University of Western Australia, Perth, Australia; 3 Wolfson Centre for Inherited Neuromuscular Diseases, Robert Jones & Agnes Hunt Orthopaedic Hospital, Oswestry, United Kingdom; 4 MRC Functional Genetics Unit, Department of Physiology, Anatomy and Genetics, University of Oxford, Oxford, United Kingdom; 5 School of Pathology and Laboratory Medicine, The University of Western Australia, Perth, Australia; 6 Neuropathology, Royal Perth Hospital and PathWest Anatomical Pathology, Perth, Australia; University of Queensland, Australia

## Abstract

Mutations in the skeletal muscle α-actin gene (*ACTA1*) cause congenital myopathies including nemaline myopathy, actin aggregate myopathy and rod-core disease. The majority of patients with *ACTA1* mutations have severe hypotonia and do not survive beyond the age of one. A transgenic mouse model was generated expressing an autosomal dominant mutant (D286G) of *ACTA1* (identified in a severe nemaline myopathy patient) fused with EGFP. Nemaline bodies were observed in multiple skeletal muscles, with serial sections showing these correlated to aggregates of the mutant skeletal muscle α-actin-EGFP. Isolated extensor digitorum longus and soleus muscles were significantly weaker than wild-type (WT) muscle at 4 weeks of age, coinciding with the peak in structural lesions. These 4 week-old mice were ∼30% less active on voluntary running wheels than WT mice. The α-actin-EGFP protein clearly demonstrated that the transgene was expressed equally in all myosin heavy chain (MHC) fibre types during the early postnatal period, but subsequently became largely confined to MHCIIB fibres. Ringbinden fibres, internal nuclei and myofibrillar myopathy pathologies, not typical features in nemaline myopathy or patients with *ACTA1* mutations, were frequently observed. Ringbinden were found in fast fibre predominant muscles of adult mice and were exclusively MHCIIB-positive fibres. Thus, this mouse model presents a reliable model for the investigation of the pathobiology of nemaline body formation and muscle weakness and for evaluation of potential therapeutic interventions. The occurrence of core-like regions, internal nuclei and ringbinden will allow analysis of the mechanisms underlying these lesions. The occurrence of ringbinden and features of myofibrillar myopathy in this mouse model of *ACTA1* disease suggests that patients with these pathologies and no genetic explanation should be screened for *ACTA1* mutations.

## Introduction

Nemaline myopathy (one of the commonest congenital myopathies) is a genetic disease of skeletal muscle classified by muscle weakness and the presence of rod-like accumulations within the myofibres called nemaline bodies (“nema” from the Greek word meaning thread; [1]). Patients usually present at birth with hypotonia, and frequently with respiratory insufficiency requiring mechanical ventilation [2]. In the most severe cases, death results within the first year of life [2]. However, adult-onset cases with minimal or mild progression also occur [3], [4].

Structurally, nemaline bodies are extensions of Z-disks and are comprised primarily of the Z-disk protein α-actinin [5], [6] but also contain myotilin [7] and sarcomeric α-actin [8]. Nemaline bodies can occur in combination with cores (core-rod myopathy; [3], [9], [10], [11], [12]), as a secondary feature in mitochondrial disorders [13] and in patients with HIV [14]. In addition, nemaline bodies can be induced experimentally [15] and sarcomeric rod-like structures form in DMSO-treated muscle cultures [16], [17].

Mutations in genes coding for six different proteins associated with muscle thin filaments have been shown to cause nemaline myopathy, namely slow muscle α-tropomyosin (*TPM3*) [18], nebulin (*NEB1*) [19], skeletal muscle α-actin (*ACTA1*) [20], troponin T1 (*TNNT1*) [21], slow muscle β-tropomyosin (*TPM2*) [22] and muscle-specific cofilin (*CFL2*) [23]. Nemaline myopathy with cores or core-like regions is caused by mutations in ryanodine receptor 1 (*RYR1*; [10]) and Kelch repeat and BTB/POZ domains-containing protein 13 (*KBTBD13;* [24]). Mutations in *NEB1* and *ACTA1* are the commonest causes of nemaline myopathy. Linkage analysis indicates that mutations in *NEB1* account for at least 50% of all nemaline myopathy patients [25] whereas mutations in *ACTA1* have been shown to cause approximately 25% of all nemaline myopathy, but 50% of the severe presentations [4]. Mutations in the other known causative genes probably contribute 5-10% of cases, with the remaining causative genes/mutations not yet identified, e.g. [26].

There are currently >180 known *ACTA1* mutations distributed through all 6 coding exons of the gene [27] (http://www.waimr.uwa.edu.au/research/lovd.html). These mutations not only produce nemaline myopathy but are also associated with additional pathological features such as intranuclear rods, actin accumulation, cores and fibre type disproportion [3], [20], [28], [29]. Most *ACTA1* mutations are missense, dominant *de novo* mutations, with the majority of patients having a severe congenital myopathy phenotype and not surviving to one year of age [27]. There is some correlation between the location of the mutated residue and the amino acid substitution (in the case of missense mutations) with the disease phenotype and severity [30]. However, due to the small number of patients with any specific mutation, the extreme disease severity and paucity of muscle biopsy material, the possibilities for investigating the pathobiology of *ACTA1* mutations using patient muscle are limited.

In order to overcome this difficulty we have investigated transgenic mouse and tissue culture models of *ACTA1* mutations. We previously generated mouse models of *ACTA1* nemaline myopathy, with the D286G mutation and showed that the disease severity correlated with the percentage of mutant protein [31]. Moreover, the vast majority of mice which expressed the ACTA1-D286G protein at ∼40% of wild-type presented with a severe splayed leg and immobility phenotype in the early postnatal period [31]. In tissue culture, transfecting C2C12 cells with mutant ACTA1-EGFP proteins in general produces similar lesions to those observed in patients' biopsies, whereas transfection with WT ACTA1-EGFP does not (e.g. [32], [33]). In our hands C2C12 cells transfected with an *ACTA1* D286G-EGFP construct produce cytoplasmic EGFP-positive aggregates in myoblasts and differentiating myotubes that are not seen in the nuclei (unpublished data), although it has been suggested that the D286G mutation does produce intranuclear rods [34].

In order to allow specific visualisation of the mutant ACTA1 D286G protein in skeletal muscle of the resulting transgenic mice, an enhanced green fluorescent protein (EGFP)-tag was included in another *ACTA1* D286G transgene construct. The transgenic C57BL/6J;CBA/Ca-Tg(ACTA1.D286G-EGFP) (abbreviated *Tg(ACTA1)* ^*D286G-EGFP*^) mice demonstrate structural abnormalities characteristic of nemaline myopathy, decreased activity and muscle weakness. Thus this mouse model displays all the hallmark features of nemaline myopathy, with the additional benefit that the mutant ACTA1 protein can be tracked in the presence of WT ACTA1 through the EGFP tag. In addition, the transgenic mice show features of myofibrillar myopathy with accumulations of granulofilamentous material by electron microscopy and desmin accumulation by immunohistochemistry and also large numbers of ringbinden and internal nuclei suggestive of degeneration and regeneration of myofibres. This mouse model thus provides a useful tool for the further study of the pathogenesis of nemaline bodies, core-like areas and ringbinden fibres and potential therapeutic interventions for these pathologies and points to other possible patient cohorts to screen for *ACTA1* mutations.

The intensity of the ACTA1-D286G-EGFP signal varied considerably not only between different muscle fibre types but with age and serves as a cautionary note to researchers using skeletal muscle promoter and enhancer constructs for expression of transgenes.

## Methods

### Generation and screening of transgenic lines

All animal procedures were approved by the Animal Experimentation Ethics Committee of The University of Western Australia (RA03/100/918).

A transgenic mouse line expressing the ACTA1 protein containing the D286G mutation, fused at the C terminus to the coding sequence for EGFP was created using standard procedures (Tinsley et al 1996). The line expressed the transgene under the control of a cassette provided by E. Hardeman (University of New South Wales, Sydney, New South Wales, Australia), containing a 2.2-kb fragment of the human skeletal muscle α-actin promoter (HSA) [35] and the troponin-I slow upstream enhancer ([36] as with our previous mouse models 31,37.

The transgenic expression construct was made as follows: The human *ACTA1* cDNA sequence (minus the stop codon) was amplified from human skeletal muscle cDNA, reverse transcribed from RNA extracted from control human skeletal muscle. The D286G mutation was introduced by site directed mutagenesis (Stratagene) and was confirmed by sequencing. Next, the *ACTA1*(D286G) sequence was inserted into the pEGFP-N1 plasmid (Clontech) before subsequent excision of the entire *ACTA1*(D286G) + EGFP sequence. A 17 amino acid linker existed between *ACTA1*(D286G) and EGFP, derived from the sequence of the pEGFP-N1 multiple cloning site.

Then, through a series of multiple sub-cloning steps, the *ACTA1* (D286G) cDNA sequence and the EGFP cDNA sequence, the 157 bp human TnI slow enhancer, and the 2.2 kb fragment of the HSA promoter, were inserted in the pcDNA3.1(+) plasmid (Invitrogen) backbone. Subsequently, the 4.5 kb combined insert, plus ∼800 bp of the bovine growth hormone poly A tail from the adjacent pcDNA3.1(+) backbone was excised as one DNA fragment and later used for injection into CBA/Ca x C57BL/6J mouse embryos by usual techniques to create the transgenic mouse line *C57BL/6J;CBA/Ca-Tg(ACTA1.D286G-EGFP)*, abbreviated to *Tg(ACTA1)^D286G-EGFP^*. Positive offspring were detected by PCR on tail-tip DNA using primers designed against the HSA promoter sequence.

### Histology and immunohistochemistry (IHC)

Freshly excised skeletal muscles were frozen in optimum cutting temperature (OCT) medium using liquid nitrogen cooled isopentane. Gomori trichrome staining was performed on 8–10 µm sections, cut on a Leica Jung CM cryocut 1800, according to standard procedures used in routine diagnostics to detect the presence of nemaline bodies [38].

For IHC, 10 μm sections were blocked for 1 hr in 1% bovine serum albumin (BSA) and 10% fetal calf serum (FCS) in phosphate buffered saline (PBS) at room temperature. Mouse IgG_1_ monoclonal antibodies against α-actinin (EA-53, diluted 1∶10, Sigma-Aldrich), MHCI (NCL-MHC1, diluted 1∶10, Novocastra), MHCIIA (SC-71, diluted 1∶3, DSHB), desmin (D033, diluted 1∶50, Dako) and αB-crystallin (1B6.1-3G4, diluted 1∶20, Abcam) were conjugated to Zenon® AlexaFluor® (Invitrogen; either 350, 488 or 594), diluted in the FCS/BSA solution and incubated overnight at 4°C. After three 5-min washes in PBS, the sections were mounted in Hydromount (National Diagnostics). The Zenon® technology prevented background staining from using mouse secondary antibodies on mouse tissue.

Mouse IgM monoclonal antibodies against MHCIIB (BF-F3, diluted 1∶10, DSHB) and MHCIIX (6H1, used neat, [39]) were incubated on sections overnight at 4°C in the BSA solution. The sections were washed three times for 5 min and incubated for 1 hr with a goat anti-mouse IgM secondary antibody (diluted 1∶1000; Alexa Fluor® 488, Invitrogen) followed by three more washes with PBS. Rabbit polyclonal anti-myotilin antibody (diluted 1∶100, [40]) was incubated on sections overnight at 4°C in the BSA solution, followed by three 5-min washes with PBS, and then a 1 hr incubation with a goat anti-rabbit secondary antibody (diluted 1∶1000; Alexa Fluor® 555, Invitrogen) followed by three 5-min washes with PBS. Appropriate isotype (mouse IgG_1_, IgM or rabbit IgG), positive and negative controls were used for each experiment. Sections were visualised using an inverted fluorescent microscope (Olympus model IX-71) and digital camera (model DP-71, Olympus) or confocal microscope (model MRC1000/1024, Biorad Laboratories Pty Ltd).

### Fibre typing and morphometry

MHC fibre-type proportions and morphometry were determined for EDL and SOL muscles from 1 and 4-month old mice as described previously [37], [41].

### Electron microscopy (EM)

Muscles were removed and immediately cut into very thin strips before immersion in 2.5% glutaraldehyde in 1 M cacodylate buffer, pH 7.4. Samples were post-fixed with 2% osmium tetroxide for 30 min, dehydrated through an ascending series of ethanols, embedded in Spurr's epoxy resin and cured for at least 3 days at 70°C. Ultrathin sections (70 nm) were cut with a LKB 8800 ultramicrotome and grids viewed and photographed with a JEOL 2100 transmission EM and 11 mega-pixel Orius digital camera.

### Skinned fibre analysis

Analysis of the contractility of mechanically-skinned EDL fibres from 6-month old *Tg(ACTA1)^D286G-EGFP^* and WT mice was performed as previously described [31], [37].

### Whole muscle contractility

The contractile properties of excised EDL and SOL muscles from 1-month old male mice were examined using an Intact Muscle Test System (Aurora Scientific Inc.) as previously described [31], [37]. Twitch force (force developed in response to a single action potential) and tetanic force response elicited in response to trains of stimulation pulses at different frequencies (10, 20, 40, 60, 80, 100 and 120 Hz) were recorded. A 3 min interval was allowed between trains of stimuli, to prevent fatigue. The tetanic responses were normalised to percentage of maximal force to examine the specific effects of stimulation frequency on force production.

Specific force (N/cm^2^) was determined using the method of [42]. At completion of functional testing, muscles were trimmed, blotted and weighed to determine muscle mass (m_m_). Muscle cross–sectional area (CSA) was determined by dividing m_m_ by the product of fibre length (L_f_) and the density of mammalian skeletal muscle [1.06 mg/mm^3^; [43]]. L_f_ was calculated by multiplying the optimal muscle length (L_o_) by a previously determined EDL L_f_-to-L_o_ ratio of 0.45 and SOL L_f_-to-L_o_ ratio of 0.71 [42]. The maximum isometric specific forces (*s*P_o_) and specific twitch forces (*s*P_t_) were then determined using the formula described in [44].

### Running wheel experiments

One-month old WT and *Tg(ACTA1)^D286G-EGFP^* mice were housed singularly in cages containing a standard mouse running wheel connected to a speedometer which recorded distance travelled, time spent moving, average and maximum speeds. Activity was monitored every 24 hours over a 7-day period and the average distance travelled per day calculated.

### Statistics

GraphPad Prism 4 was used to conduct two-tailed t tests with Welch's correction for all the statistical analyses, except for the force-frequency datasets. Multivariate analyses of variance were conducted using Statistica (StatSoft Pacific Pty Ltd) with main effects of genotype and repeated measures of frequency. When the ANOVA indicated significant main effects and interaction, individual means were compared with a Student–Newman–Keuls post hoc test. All data are presented as the mean ± standard error of the mean.

## Results

### *Tg(ACTA1)^D286G-EGFP^* mice developed rod-like bodies

**Figure 1:**
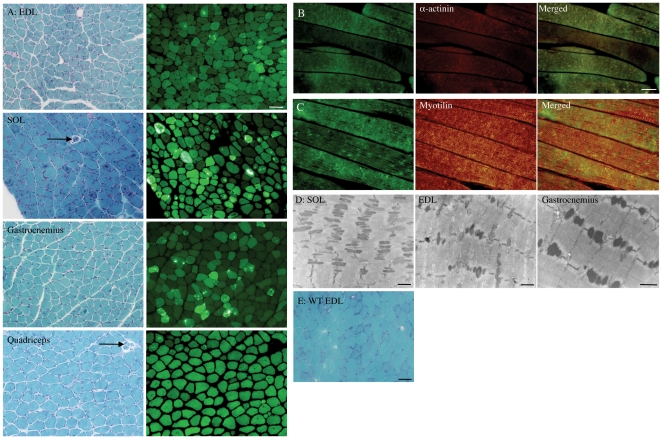
Nemaline bodies were a prominent feature of *Tg(ACTA1)^D286G-EGFP^* muscle. (A) Gomori trichrome staining revealed that nemaline bodies (see insert) were a prominent feature of *Tg(ACTA1)^D286G-EGFP^* muscles, especially at 1 month of age. Serial sections showed that the location of these nemaline bodies corresponded with regions of intense EGFP aggregates. Note also the presence of internal nuclei, the large variation in fibre size in muscle of *Tg(ACTA1)^D286G-EGFP^* mice and the absence of EGFP signal in spindle fibres (arrows). These first two characteristics were not observed in WT muscle (Figure 1.E: WT EDL muscle stained with Gomori trichrome). ACTA1(D286G)-EGFP α-actin was incorporated into the sarcomeres as seen by the striated EGFP signal but also formed intensely fluorescing aggregates in quadriceps muscles (B, C). The EGFP-positive aggregates labeled with antibodies to the Z-disk proteins α-actinin (B) and myotilin (C). (D) Electron-dense nemaline bodies were observed as extensions of the Z-disk. These were most frequently observed in the SOL muscle of 1-month old and the EDL and gastrocnemius muscles of 4-month or older *Tg(ACTA1)^D286G-EGFP^* mice. Scale bars  =  50 µm (A, E), 25 µm (B, C) and 2 µm (D).

Gomori trichrome staining revealed intense red-staining structures similar to nemaline bodies as seen in nemaline myopathy patients and serial sections showed that EGFP-positive accumulations correlated with the location of these in the EDL, SOL, gastrocnemius and quadriceps muscles (Figure 1.A). Quadriceps muscle from 4-month old mice expressing the ACTA1(D286G)-EGFP fusion protein contained numerous EGFP-positive accumulations and rod-like structures (Figures 1.B and C). The majority of these aggregates also co-stained with antibodies to the Z-disk proteins α-actinin (Figure 1.B) and myotilin (Figure 1.C), both proteins shown to be present in nemaline bodies. At the ultrastructural level, electron-dense nemaline bodies were observed in various muscles from *Tg(ACTA1)^D286G-EGFP^* mice (Figure 1.D) suggesting that the red-staining structures visualised by Gomori trichrome were indeed nemaline bodies. Thickened Z-disks and extensions of the Z-disks were also observed in *Tg(ACTA1)^D286G-EGFP^* muscle by EM (Figure 1.D).

### Muscle physiology

**Figure 2:**
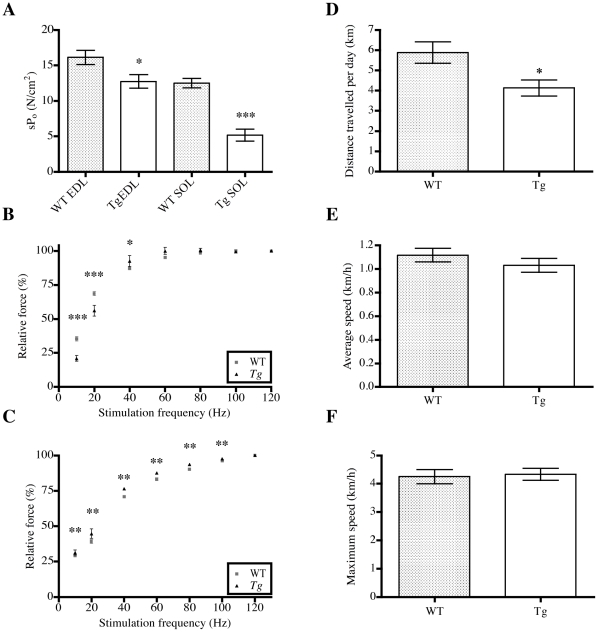
*Tg(ACTA1)^ D286G-EGFP^* mice were weak and less active at 1 month of age than WT control mice. (A) *s*P_o_ was significantly lower in EDL (*n = 9, p = 0.026*) and SOL muscles (*n = 10, p<0.0001*) of *Tg(ACTA1)^D286G-EGFP^* mice compared to that of WT muscle (EDL: *n = 11*, SOL: *n = 8*). (B) In the SOL muscles from *Tg(ACTA1)^D286G-EGFP^* mice, the force–frequency relationship displayed a significant shift to the right compared to WT. (C) The force-frequency relationship was shifted to the left in the *Tg(ACTA1)^D286G-EGFP^* EDL muscles compared to WT EDL (NOTE: for some data points the SEM bars are too small to be seen). (D) One-month old *Tg(ACTA1)^D286G-EGFP^* mice (*n = 8*) used voluntary running wheels less than WT mice (*n = 9*), and traveled 30% less distance on average, per day. (E) Average and (F) maximum speeds were not different between *Tg(ACTA1)^D286G-EGFP^* and WT mice. *p<0.05, **p = 0.005, ***p<0. 001.

To investigate the functional consequences of the mutant skeletal muscle α-actin and associated structural abnormalities, the contractile properties of isolated EDL and SOL muscles of 1-month old WT and *Tg(ACTA1)^D286G-EGFP^* mice were examined. Maximal specific force was significantly decreased in both EDL (∼79% of WT; *p = 0.026*) and SOL muscles of the *Tg(ACTA1)^D286G-EGFP^* mice, with the SOL most greatly affected (∼42% of WT; *p<0.0001*; Figure 2.A). In addition, the force-frequency relationship of *Tg(ACTA1)^D286G-EGFP^* SOL muscles exhibited a marked shift to the right compared to WT (Figure 2.B), indicating that a greater stimulation frequency was required in *Tg(ACTA1)^D286G-EGFP^* SOL muscles to produce a similar amount of force to WT. Conversely, the force-frequency relationship of *Tg(ACTA1)^D286G-EGFP^* EDL muscle displayed a small, but significant shift to the left compared to WT EDL muscles (Figures 2.C). The peak specific twitch force (*s*P_t_) was significantly diminished in *Tg(ACTA1)^D286G-EGFP^* SOL muscles compared to WT, but there was no significant difference in *s*P_t_ between *Tg(ACTA1)^D286G-EGF^* and WT EDL muscles (Table 1). No significant difference in the contraction or relaxation times of the twitch responses was found in the EDL or SOL muscles of *Tg(ACTA1)^D286G-EGFP^* and WT mice (Table 1). These results indicate that the actin mutation in *Tg(ACTA1)^D286G-EGFP^* mice significantly alters skeletal muscle contractile performance, and this effect is especially pronounced in the predominantly slow twitch SOL muscle.

**Table 1:** Contractile properties of twitch responses elicited in EDL and SOL muscles from *Tg(ACTA1)^D286G-EGFP^* and WT mice.

*Table 1:* * Contractile properties of twitch responses*			
	WT	*Tg(ACTA1)^D286G-EGFP^*	*p-value*
***s*** **P_t_ (N/cm^2^)**			
EDL	4.28±0.31 (*12*)	3.43±0.35 (*9*)	*0.085*
SOL	2.13±0.14 (*8*)	0.411±0.14 (*9*)	*<0.0001*
**TTP (ms)**			
EDL	22.3±0.5	23.1±0.5	*0.255*
SOL	41.9±1.2	40.1±2.4	*0.522*
**½ RT (ms)**			
EDL	29.1±2.1	29.6±2.3	*0.889*
SOL	55.8±4.8	50.2±6.6	*0.509*

Data are presented as mean ± SEM, values in parenthesis represents the *n* of isolated muscles used per group.

### Behavioural analysis

Voluntary running wheel activity of 1-month old *Tg(ACTA1)^D286G-EGFP^* mice was significantly reduced over a seven-day period. The average distance travelled per day by the *Tg(ACTA1)^D286G-EGFP^* mice was ∼30% less than that of WT mice (*p = 0.02;* Figure 2.D). However average and maximum speeds were similar for *Tg(ACTA1)^D286G-EGFP^* and WT mice (Figures 2.E and F).

### Transgene protein levels varied in different muscles at different ages and in different fibre types

**Figure 3:**
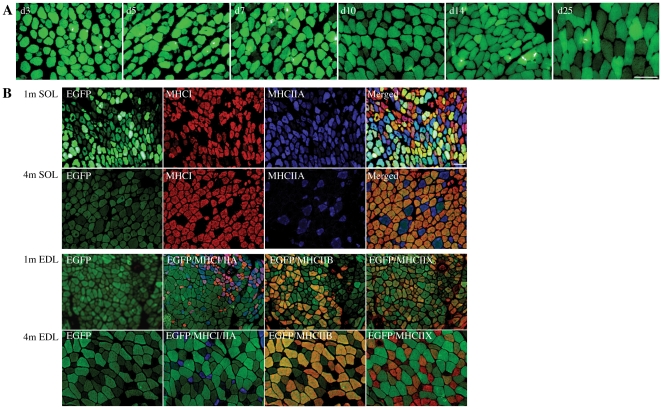
Expression of the *ACTA1*(D288G)-EGFP fusion α-actin protein changes with MHC fibre type and was associated with an increased contribution of MHC hybrid fibres. (A) A time-course of EGFP-expression in the gastrocnemius muscle indicated that during the early PN period all fibres expressed similar levels of *ACTA1*(D288G)-EGFP. By PN d7 expression was slightly reduced and by PN d25 a proportion of the fibres expressed comparatively low levels of *ACTA1*(D288G)-EGFP. (B) IHC of serial sections of SOL and EDL muscles from 1- and 4-month old *Tg(ACTA1)^D286G-EGFP^* mice revealed that in the SOL, the fibres expressing the most *ACTA1*(D288G)-EGFP were MHCI and MHCI/IIA hybrids (1 month) or pure MHCI fibres (4 months). In the EDL, the EGFP signal was most intense in the MHCIIB fibres for both timepoints. By 4 months of age in the EDL, MHCIIA fibres were essentially negative for EGFP by comparison to MHCIIB and MHCIIX fibres. Scale bar  =  50 µm. Note the EGFP images were not all taken at the same exposure times for gastrocnemius, EDL and SOL muscles but were instead taken at the settings required to achieve sufficient EGFP signal to facilitate visualisation of the fibres.

**Figure 4:**
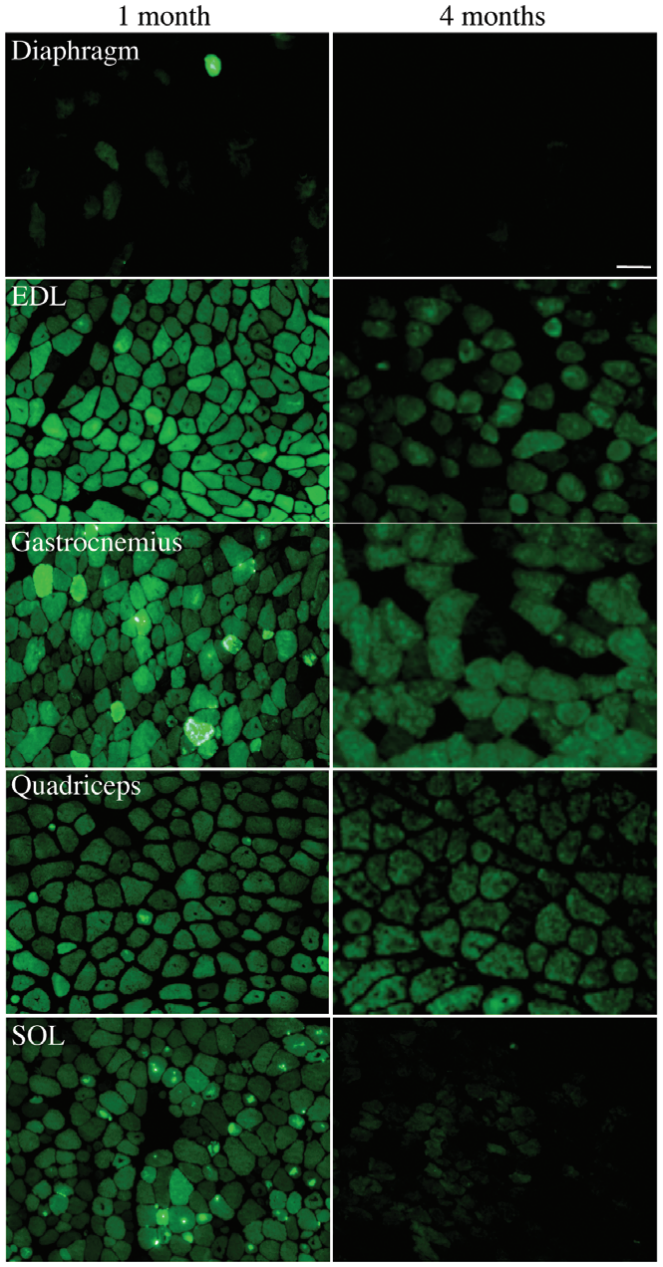
The EGFP signal intensity varied between muscles and diminishes with age. The EGFP signal imaged under the same exposure settings clearly demonstrated that the amount of mutant skeletal muscle α-actin-EGFP varied between different muscles, with intense EGFP aggregates most frequent in the EDL, gastrocnemius and SOL muscles at 1 month of age. By 4 months of age the intensity of the EGFP signal was reduced in all muscles compared to the 1-month time point. This was most noticeable in the SOL muscle. The diaphragm was largely EGFP-negative at both time-points. Scale bar  =  50 µm.

The intensity of the EGFP-signal (reflective of the amount of mutant skeletal muscle α-actin protein present), and the extent of structural abnormalities varied considerably with age and across different muscles (Figs 3 and 4). During the early PN period (PN d3 and d5) all limb muscle fibres examined showed similar levels of the EGFP fusion protein (Figure 3.A). However, by PN d7 there was variation in the EGFP signal intensity in different muscle fibres, with this variation becoming more pronounced with PN development, such that at PN d25 there were clearly two populations of fibres: high and low EGFP-expressors. At 1 month of age, levels of the transgenic protein in the SOL were greater in MHCI and MHCI/IIA hybrid fibres, whereas by 4 months of age levels were was negligible in MHCIIA fibres and low in MHCI fibres and considerably less than in EDL MHCIIB and IIX fibres (Figure 3.B). By 4 months of age in the EDL, the ACTA1(D286G)-EGFP protein became confined to predominantly MHCIIB and MHCIIX fibres and was absent from MHCIIA fibres (Figure 3.B). This fibre-type dependent expression of the transgene is likely to be at least partially responsible for the altered EGFP signal intensities observed between different muscles and at different ages (Figure 4), with levels of the ACTA1(D286G)-EGFP protein high in all muscles at the younger timepoint of 1 month compared to 4 months. The diaphragm of *Tg(ACTA1)^D286G-EGFP^* expressed very little EGFP at all ages examined. Interestingly, mutant protein was never detected in the intrafusal fibres within the muscle spindles (Figure 1.A).

**Figure 5:**
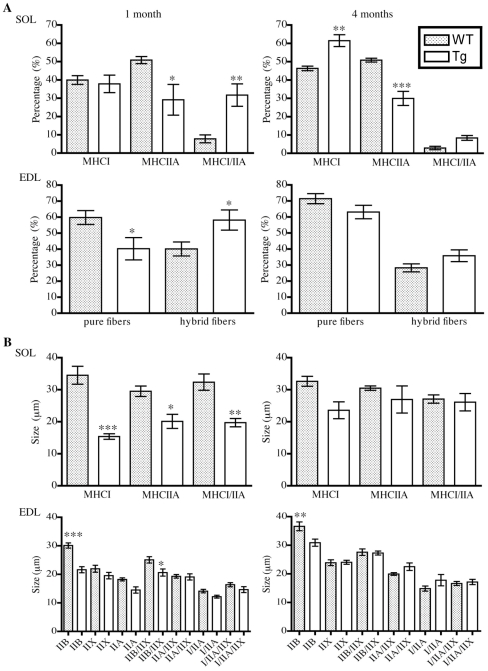
Fibre-type proportions and fibre size were significantly different in *Tg(ACTA1)^D286G-EGFP^* muscle. (A) At 1 month of age, *Tg(ACTA1)^D286G-EGFP^* SOL muscle (*n = 8*) contained significantly less MHCIIA fibres and a concurrent increase in the percentage of MHCI/IIA hybrid fibres than in 1-month old WT SOL muscle (*n = 9*). This change persisted at 4 months, and in addition the *Tg(ACTA1)^D286G-EGFP^* SOL (*n = 5*) also contained a higher percentage of MHCI fibres than WT SOL (*n = 5*). At 1 month of age a significant increase in the percentage of hybrid fibres was observed in *Tg(ACTA1)^D286G-EGFP^* EDL muscle (*n = 7*) compared to WT EDL (*n = 10*). By 4 months of age the contribution of hybrid versus pure fibres was not significantly different for WT (*n = 6*) and *Tg(ACTA1)^D286G-EGFP^* (*n = 5*) EDL muscle. (B) Morphometry of EDL and SOL muscle fibres at 1 and 4 months of age revealed that there is a trend towards all *Tg(ACTA1)^D286G-EGFP^* fibres being smaller than WT. This feature was more striking at the 1-month time-point for both the EDL and SOL fibres. *p<0.05, **p<0.01, ***p<0.001 compared to WT.

Coupled with these alterations in the level of the ACTA1(D286G)-EGFP α-actin with fibre type and age was a shift in the proportions of muscle fibre types in the EDL and SOL muscles in the transgenic mice compared to WT mice (Figure 5). In the *Tg(ACTA1)^D286G-EGFP^* EDL muscle at 1 month of age there was a significant reduction in the proportion of pure fibres (*p = 0.024*) with a concurrent increase in the proportion of hybrid fibres expressing more than one MHC isoform (Figure 5.A, *p = 0.028*). All fibre types were smaller in *Tg(ACTA1)^D286G-EGFP^* EDL than in WT muscle, however the fibre size difference was only significant for pure MHCIIB (*p<0.001*) and hybrid MHCIIB/IIX fibres (*p<0.05*, Figure 5.B). At 4 months of age no differences in the EDL fibre type proportions were observed compared to WT muscle and only pure MHCIIB fibres (the muscle fibres with the highest expression of the transgene) were significantly smaller (*p<0.01*). At 1 month, *Tg(ACTA1)^D286G-EGFP^* SOL muscle contained a reduced proportion of MHCIIA (*p = 0.018*) and an increased contribution of MHCI/IIA hybrid fibres (Figure 5.A, *p = 0.002*), with all fibre types significantly smaller than WT fibres (Figure 5.B). The decreased contribution of MHCIIA fibres observed in the 1-month old SOL muscle of *Tg(ACTA1)^D286G-EGFP^* mice persisted at 4 months of age (*p<0.001*) and there was also a significant increase in the percentage of pure MHCI fibres (*p = 0.002*).

### *Tg(ACTA1)^D286G-EGFP^* muscles demonstrate other pathologies: internal nuclei, core-like areas, granulofilamentous electron dense inclusions and ringbinden

**Figure 6:**
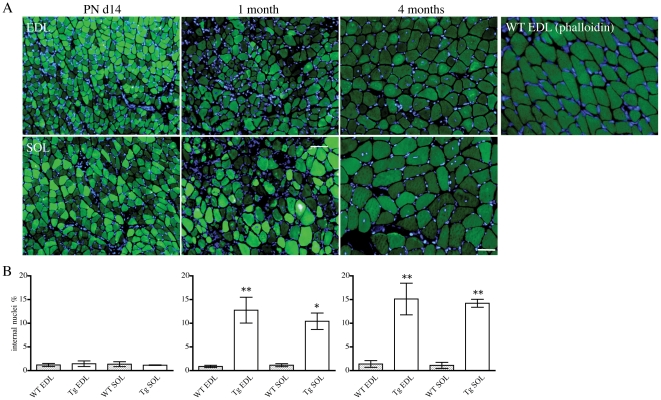
Internally-nucleated fibres were a persistent feature of skeletal muscle from *Tg(ACTA1)^D286G-EGFP^* mice post-weaning. (A) Representative images taken of Hoechst-labeled EDL and SOL muscles from PN d14, 1 and 4-month old *Tg(ACTA1)^D286G-EGFP^* mice showed the occurrence of internally-nucleated fibres in post-weaned *Tg(ACTA1)^D286G-EGFP^* mice. A representative image of a WT EDL muscle labelled with phalloidin-FITC and Hoechst is also shown. (B) The percentage of internally-nucleated fibres in whole EDL (1m, 4m WT: *n = 7*, *n = 6*; *Tg*: *n = 10*, *n = 5*) and SOL (1m, 4m WT: *n = 9*, *n = 5*; *Tg*: *n = 8*, *n = 5*) muscles was determined for WT and *Tg(ACTA1)^D286G-EGFP^* mice, with a significant increase seen in *Tg(ACTA1)^D286G-EGFP^* muscle. However there was no significant difference in the number of internally nucleated fibres in EDL and SOL muscles (WT: *n = 7*, *Tg*: *n = 4*) of *Tg(ACTA1)^D286G-EGFP^* mice at PN d14. *p<0.05 compared to WT, **p<0.01 compared to WT.

**Figure 7:**
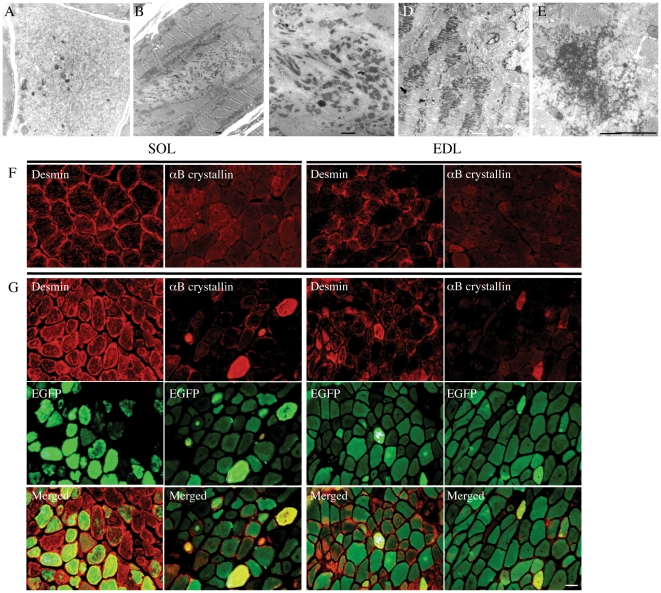
SOL muscle of 1-month old *Tg(ACTA1)^D286G-EGFP^* mice displayed, in addition to nemaline bodies, a number of alterations at the ultrastructural level that are found in rod-core and myofibrillar myopathies. (A) Aggregates of sarcoplasmic reticulum profiles punctuated by scattered autophagosomes, mitochondria and intermediate filaments. (B) An intermyofibrillar aggregate of fine filaments and electron dense rods of varying dimensions. (C) A collection of anastomosing, irregular electron dense rods into which fine filaments are inserting. The electron dense rods have a faintly fibrillar substructure. (D) Fibrillogranular electron dense material accumulating along the plane of the Z-bands. Note that the Z-bands are fragmented and there is disorganisation of the sarcomeric myofilaments. (E) Transverse view of electron dense fibrillogranular material. Labelling of SOL muscle sections from *Tg(ACTA1)^D286G-EGFP^* mice revealed altered localisation and expression levels of desmin and αB-crystallin (G) compared to that seen for WT muscle (F). Desmin expression was more diffuse throughout the muscle fibres in *Tg(ACTA1)^D286G-EGFP^* muscle compared to WT muscle. Fibres containing intense EGFP-positive aggregates also showed elevated levels of αB-crystallin compared to neighbouring fibres. Scale bars  =  2 µm (A-E), 20 µm (F–G).

**Figure 8:**
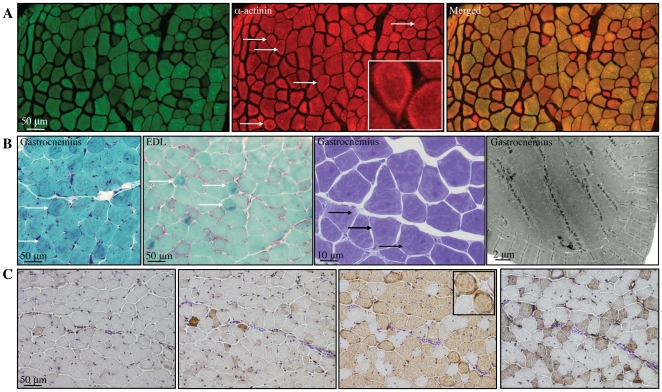
Ringbinden fibres were a prominent feature of skeletal muscle from adult *Tg(ACTA1)^D286G-EGFP^* mice. (A) Confocal imaging of EDL muscle from a 4-month old *Tg(ACTA1)^D286G-EGFP^* mouse showed the presence of numerous ringbinden fibres (arrows) that were best seen when labelled with an antibody to α–actinin. (B) Ringbinden fibres were most frequent in EDL and gastrocnemius muscles of adult *Tg(ACTA1)^D286G-EGFP^* mice (≥ 4-months old) and were prominent by Gomori trichrome and toluidine blue staining (arrows). At the ultrastructural level, ringbinden fibres consisted of 2 to 7 peripheral myofibrils arranged concentrically around the central myofibres. (C) Fibre-typing of gastrocnemius muscle using DAB of consecutive sections, revealed that the ringbinden fibres were exclusively MHCIIB fibres.

In addition to the presence of nemaline bodies, EM revealed that a number of different pathological features were present in skeletal muscle of the *Tg(ACTA1)^D286G-EGFP^* mice, including internal nuclei (Figure 6) core-like regions, cores containing rods, granulofilamentous electron-dense inclusions (Figure 7) and ringbinden (Figure 8).

At PN d14 the percentage of internally nucleated fibres in the EDL and SOL was similar for WT and *Tg(ACTA1)^D286G-EGFP^* muscle at ∼1%. However, the number of internally-nucleated fibres was elevated in both EDL and SOL muscles of *Tg(ACTA1)^D286G-EGFP^* mice compared to WT mice at 1 and 4 months of age with up to ∼15% of muscle fibres demonstrating internal nuclei (Figures 6.A and B). Internal nuclei were also a prominent feature of the quadriceps and gastrocnemius of *Tg(ACTA1)^D286G-EGFP^* mice older than 1 month and were present in fibres of all MHC types (data not shown).

Core-like regions, cores containing rods and granulofilamentous electron-dense inclusions were most common in the SOL muscle of 1-month old *Tg(ACTA1)^D286G-EGFP^* mice (Figures 7.A-E). As granulofilamentous inclusions are frequently observed in myofibrillar myopathy (MFM) [45], the localisation of MFM-associated proteins was examined. Muscle samples from 1-month old *Tg(ACTA1)^D286G-EGFP^* mice were demonstrated to contain accumulations of desmin and αB-crystallin that were not seen in WT muscle (Figures 7.F and G).

The presence of ringbinden was a prominent feature of muscle from adult *Tg(ACTA1)^D286G-EGFP^* mice. By light microscopy, these fibres were best observed with Gomori trichrome staining and α-actinin immunostaining (Figures 8.A and 8.B). They were frequently observed in the EDL, gastrocnemius and quadriceps muscles of adult *Tg(ACTA1)^D286G-EGFP^* mice. In the EDL muscle of 4 month old *Tg(ACTA1)^D286G-EGFP^* mice approximately 9% of fibres were ringbinden (*n = 5*). By EM these fibres contained aggregates of 2 to 7 myofibrils arranged concentrically around the bulk of longitudinally orientated myofibrils (Figure 8.B). Fibre typing by IHC for specific MHC isoforms, performed on serial sections of gastrocnemius muscle, revealed that the ringbinden were exclusively MHCIIB fibres, though not all MHCIIB fibres were ringbinden (Figure 8.C). Ringbinden were never observed in SOL muscle, which comprises only MHCI and MHCIIA fibres, or in the other muscles, EDL, gastrocnemius and quadriceps at the earlier, 1 month, timepoint.

## Discussion

### *Tg(ACTA1)^D286G-EGFP^* mice model nemaline myopathy

Gomori trichrome staining of serial sections revealed the presence of nemaline bodies within different muscles of *Tg(ACTA1)^D286G-EGFP^* mice. These nemaline bodies correlated with strong EGFP signals indicating that the nemaline bodies likely contained the ACTA1(D286G)-EGFP protein (Figure 1.A). Skeletal muscle of *Tg(ACTA1)^D286G-EGFP^* mice contained numerous aggregates of the mutant fusion protein that co-labelled with the Z-disk proteins α-actinin and myotilin (Figures 1.B and C) - which both label nemaline bodies in patient biopsies [5], [6], [7]. At the ultrastuctural level, numerous electron-dense nemaline bodies were observed as extensions of the Z-disk in the SOL, EDL and gastrocnemius muscles of different aged *Tg(ACTA1)^D286G-EGFP^* mice (Figure 1.D). Thus, the *Tg(ACTA1)^D286G-EGFP^* mouse model displays the pathological hallmarks of nemaline myopathy. Contemporaneous to the peak occurrence of structural lesions at 1 month of age, 1-month old *Tg(ACTA1)^D286G-EGFP^* mice also exhibited reduced voluntary activity on running wheels (Figure 2.D) and isolated muscles were significantly weaker than those of WT mice. The muscle weakness was more pronounced in the muscle with the most severe structural abnormalities, i.e. 1 month SOL muscle (Figures 2.A). The age-dependent features correlated with the levels of the ACTA1(D286G)-EGFP protein present over time. Taken together, the *Tg(ACTA1)^D286G-EGFP^* mouse line is an excellent model of both the characteristic pathological lesions and the muscle weakness of nemaline myopathy, similarly to our *Tg(ACTA1)^D286G^* models [31].

A previous study showed that skeletal muscles from a mouse expressing EGFP alone (driven by the β-actin promoter) are fluorescent green and have “very high levels of GFP expression”, however they do not report any kinds of skeletal muscle pathology due to this EGFP expression, and there was no mention of any rod-like bodies. Thus the presence of EGFP rod-like bodies in our model is unlikely to be due simply to high levels of the ACTA1(D286G)-EGFP protein [46].

### Transgenic protein levels and structural lesions varied in different muscles, muscle fibre types and ages

Levels of the mutant protein varied between different skeletal muscles and at different ages, as highlighted in Figures 1, 3 and 4. During the early PN period the mutant protein was present at similar levels in all myofibres (until approximately PN d7) and then diminished in some fibre types more than others (Figure 3.A). The fibres that were brightest for EGFP in the 1- and 4-month old EDL muscle biopsies were MHCIIB-positive (Figure 3.B), with the mutant protein remained at high levels in MHCIIB fibres at 4 months of age and in MHCIIX fibres only at lower levels.

Previous studies have reported that the human skeletal muscle α-actin (HSA) promoter produces a fibre-type dependent expression of the transgene, with greatest expression in MHCIIB fibres 47,48. Preferential effects of transgenes in fast glycolytic fibres has previously been reported for other muscle gene promoters, including the muscle creatine kinase promoter [49] and the myosin light chain promoter [50]. Our current study using the easily-visualised EGFP-tagged skeletal muscle α-actin has highlighted the large variations and transient nature of transgene expression under the control of the HSA promoter and TnI(slow) enhancer in different muscle fibre types and the potential pitfalls of interpretation of mouse models generated using these promoter constructs. For example, the result clarifies an effect we have seen in the *Tg(ACTA1)^D286G+/+^* mouse model lacking the EGFP tag [31] where further (unpublished) studies have shown that the percentage of mutant protein, as determined by mass spectrometry, was similar in 1- and 4-month old gastrocnemius muscles but was reduced by ∼80% in 4-month old SOL muscles compared to 1-month old SOL. Recently, Tai *et al.* 2011, identified the modulatory region 1 in intron 1 of the muscle creatine kinase gene as enhancing MCK promoter construct expression in slow, type I and fast type IIA fibres [51]. Similar regions must exist for the other muscle genes but have not yet been precisely identified [35].

### Pathophysiology – disease mechanisms

Although differences in the extent of the pathological lesions within muscle from the *Tg(ACTA1)^D286G-EGFP^* mice might be presumed to reflect the altered physical demands and stresses placed on each, the degree of structural abnormalities does not appear to correlate with muscle use since the diaphragm is relatively unaffected even at PN d14 (data not shown, Figure 4). A similar finding was reported for the Met9Arg *TPM3* nemaline myopathy transgenic mouse [48]. In the *Tg(ACTA1)^D286G-EGFP^* mice, the SOL muscle comprises a similar MHC isoform composition to the diaphragm, thus a high percentage of MHCIIA fibres cannot alone account for the relative absence of ACTA1(D286G)-EGFP α-actin protein and EGFP-aggregates in the diaphragm. This finding possibly indicates that physiotherapy, exercise and related activities may not induce muscle damage in certain congenital myopathy patients. A study of the Met9Arg *TPM3* transgenic congenital myopathy mouse revealed that disuse-induced muscle weakness and nemaline body formation could be mitigated with exercise [52]. At the clinical level there is a growing body of anecdotal evidence to suggest that exercise may be beneficial for patients with congenital myopathies, including *ACTA1* congenital myopathies [53], [54].

In this study, the EDL and SOL muscles from *Tg(ACTA1)^D286G-EGFP^* mice were significantly weaker than WT at 1 month of age. These findings are likely because of decreased numbers of functional sarcomeres, due to the presence of non-contractile areas such as nemaline bodies and core-like regions. The 1 month SOL muscles had a greater percentage reduction in maximum force compared to EDL muscles, consistent with the greater incidence of (non-contractile) core-like regions and granulofilamentous electron dense inclusions in SOL fibres compared to other *Tg(ACTA1)^D286G-EGFP^* muscles (Figure 7.A).

In 1-month old *Tg(ACTA1)^D286G-EGFP^* mice the percentage of internally nucleated fibres was increased 15- and 9-fold compared to WT mice in EDL and soleus muscles, respectively. This difference persisted at 4 months of age (Figures 6.A and B). Despite the fact that Met9Arg *TPM3* nemaline myopathy mice are reported to have delayed maturation on the basis of perturbed muscle fibre-type proportions, internally-nucleated fibres have not been observed [48], [52], [55]. Internal nuclei are associated with degeneration and regeneration of skeletal muscle fibres [56] and as such are a characteristic feature of dystrophic muscle. They are not however, usually a feature of nemaline myopathy; therefore it is of interest that internal nuclei were present in a *Tg(ACTA1)^D286G-EGFP^* muscle. It is well known that the *mdx* mouse (model of Duchenne muscular dystrophy) undergoes a period of extensive degeneration and regeneration coincidental with weaning (∼ PN d21), which is thought to be a result of increased motor activity [57]. Thus the observed abundance of internally nucleated fibres at 1 month of age (but not PN d14), in the *Tg(ACTA1)^D286G-EGFP^* mice may be due to a similar trigger to that in the *mdx* mouse, and this feature persisted at 4 months of age.

It has been demonstrated by *in vitro* studies that certain *ACTA1* mutations induce cell death [34], [58], [59]. The mutations studied in Wallefeld *et al*. (2006) affect the normal termination codon, and as a result part of the normal 3′-UTR is translated, resulting in a larger actin protein. The clinical phenotype of all patients identified to date with such mutations is severe, with an atypical markedly dystrophic phenotype consisting of loss of muscle fibres and fibrosis seen on muscle biopsy [58]. The actin protein produced by the ACTA1(D286G)-EGFP transgene is similar in having the EGFP C-terminal tag increasing the size of the actin protein. Akin to the human patients with mutations of their *ACTA1* stop codon, the *Tg(ACTA1)^D286G-EGFP^* mice have relatively severe muscle pathologies despite low levels of mutant protein in their muscle fibres.

### Fibre type composition and fibre sizes were altered in *Tg(ACTA1)^D286G-EGFP^* mice

Coupled to the alterations in the fibre-type specific expression of the transgene with development was an alteration in the fibre type proportions in *Tg(ACTA1)^D286G-EGFP^* muscle compared to WT muscle (Figure 5). In the *Tg(ACTA1)^D286G-EGFP^* EDL muscle at 1 month of age a significant reduction in the proportion of pure fibres was observed, with a concurrent increase in the proportion of hybrid fibres and a decrease in the fibre diameter of all fibres. Conversely by 4 months of age no significant differences in fibre type proportions in EDL were detected compared to WT muscle. At 1 month of age *Tg(ACTA1)^D286G-EGFP^* SOL muscles contained a reduced proportion of pure MHCIIA and an increased contribution of MHCI/IIA hybrid fibres, with all fibre types being significantly smaller than those of WT mice. The decreased contribution of MHCIIA fibres observed in the 1-month old SOL muscle of *Tg(ACTA1)^D286G-EGFP^* mice persisted at 4 months of age, however there was also a significant increase in the percentage of pure MHCI fibres. This indicates that the elevated number of MHCI/IIA hybrid fibres observed at 1 month of age had perhaps with time converted into pure MHCI fibres in the *Tg(ACTA1)^D286G-EGFP^* SOL muscle. Thus, the presence of an increased proportion of hybrid fibres in *Tg(ACTA1)^D286G-EGFP^* muscle at 1 month of age, likely reflects muscle degeneration/regeneration and is consistent with the occurrence of elevated numbers of internally-nucleated fibres post-weaning in the *Tg(ACTA1)^D286G-EGFP^* muscle (Figure 6).

### Structural lesions other than nemaline bodies

A number of pathological features in addition to the classical nemaline bodies were observed in *Tg(ACTA1)^D286G-EGFP^* muscle by EM, including core-like regions and granulofilamentous electron dense inclusions (Figure 7.A). These features were most frequently observed in the SOL muscle of 1-month old *Tg(ACTA1)^D286G-EGFP^* mice. To further examine the nature of these abnormalities, IHC was performed for proteins that have been shown to comprise core-like regions and osmiophilic inclusions. Antibodies to desmin and αB-crystallin demonstrated abnormal staining in muscle from 1-month old *Tg(ACTA1)^D286G-EGFP^* mice (Figures 7.B and C). The desmin staining pattern was more diffuse in the *Tg(ACTA1)^D286G-EGFP^* muscle and bright desmin-positive regions that were devoid of *ACTA1*(D286G)-EGFP α-actin were observed. αB-crystallin staining was elevated in *Tg(ACTA1)^D286G-EGFP^* fibres that showed intense *ACTA1*(D286G)-EGFP staining. These ultrastructural abnormalities and abnormal desmin and αB-crystallin staining are the hallmarks of myofibrillar myopathy (MFM). Slightly abnormal desmin and αB-crystallin staining was also previously observed in *Tg(ACTA1)^D286G^* fibres, but evidence of osmiophilic inclusions were not seen by EM [31]. Cross sections of the diaphragm from a patient with this mutation were stated to contain bright eosinophilic inclusions which is not the usual staining of nemaline bodies and is more indicative of MFM, but the biopsy and autopsy material are no longer available for study (Alan Beggs and Caroline Sewry, personal communication). This finding suggests that patients with MFM of unknown genetic origin should perhaps be screened for *ACTA1* mutations. Such a strategy might also be suggested from the finding that abnormalities of actin localisation are seen in the muscle fibres of MFM patients [60].

### Ringbinden are a prevalent feature of Tg(ACTA1)^D286G-EGFP^ muscle

Ringbinden are a frequent feature of muscular dystrophies and myopathies [61], as well as being prevalent in muscles which do not attach from bone to bone (e.g. the diaphragm and extraocular muscle [62]). Studies of regenerating and/or tenotomised muscle [63], [64] or muscle prone to myotendinous junction disruption [65] have shown that ringbinden fibres can be experimentally induced. The presence of ringbinden in *Tg(ACTA1)^D286G-EGFP^* EDL, gastrocnemius and quadriceps muscles at 4 months of age (Figure 8), but not 1 month of age, may be reflective of an abnormal repair process or a protective mechanism. In our previous study of *Tg(ACTA1)^D286G^* mice, expressing the same mutated actin (D286G) as in the present study but lacking the C-terminal EGFP tag, we also only observed ringbinden in type IIB fibres of 4-month or older *Tg(ACTA1)^D286G^* mice. Ringbinden were not present in muscle of *Tg(ACTA1)^D286G^*. *Acta1^+/-^* pups despite the disease severity and greater percentage of mutant protein [31]. This is in keeping with the hypothesis that ringbinden may arise due to an abnormal repair process over time rather than due to the extent of muscle weakness or structural lesions [65].

In conclusion, the *Tg(ACTA1)^ D286G-EGFP^* mouse models nemaline myopathy both in terms of muscle weakness and abnormal morphology, and as such could be used to understand better the pathobiology of the disease as well as serve as a useful animal model in which to test possible therapies. Due to the EGFP tag, this model is uniquely placed to specifically track the location of the mutant ACTA1 protein in the presence of WT ACTA1. The altering expression of both the ACTA1-D286G-EGFP and ACTA1-D286G transgenes provides a cautionary tale to those researchers using similar expression constructs to mediate expression of transgenes. Furthermore, the various structural lesions (core-like regions, osmiophilic inclusions and ringbinden fibres), observed in these mouse models, support the proposal that the various pathological features observed in biopsies from *ACTA1* patients represent a continuum of disease features rather than discrete pathological entities. Finally, patients with large numbers of ringbinden-containing fibres and/or myofibrillar myopathy pathologies and no molecular explanation of their diseases should probably be tested for *ACTA1* mutations.
